# Knowledge and Attitudes of Cypriots on Melanoma Prevention: Is there a Public Health Concern?

**DOI:** 10.1186/s12889-021-12324-0

**Published:** 2022-01-08

**Authors:** Demetra Kyprianou, Iris Charalambidou, Oluwaseun Famojuro, Hongmei Wang, Dejun Su, Paraskevi A. Farazi

**Affiliations:** 1 Department of Life and Health Sciences, University of Nicosia, Nicosia, Cyprus; 2 Department of Epidemiology, College of Public Health, University of Nebraska Medical Center, Omaha, NE 68198-4395 USA; 3 Department of Health Services Research & Administration, College of Public Health, University of Nebraska Medical Center, Omaha, NE USA; 4 Department of Health Promotion, College of Public Health, University of Nebraska Medical Center, Omaha, NE USA

**Keywords:** Melanoma, knowledge, climate change, Cyprus

## Abstract

**Background:**

Melanoma is the deadliest type of skin cancer. It is the eighth most common cancer in males and the tenth in females in Cyprus, an island in the Mediterranean with a high ultraviolet (UV) index. Cyprus is expected to be strongly affected by climate change and consequently, melanoma will likely become an increasing public health problem. Melanoma prevention is possible; however, it is unknown if people living in Cyprus are aware of melanoma and prevention methods. To this end, we used a validated survey to evaluate the level of melanoma knowledge and factors associated with it in the Cypriot population.

**Methods:**

We conducted a 47-item survey with sections on demographics, knowledge of melanoma and risk factors, attitudes toward relevant health practices, and protective behaviors among six hundred Cypriot residents from October 2015 to April 2016.

**Results:**

Our results revealed that only 59% of participants check their skin for moles, 87% protect their skin from the sun during summer holidays, and 57% do not take measures to protect their skin from the sun during non-holiday periods. Protective behavior was positively associated with educational level (*P*=0.016) and district of residence (*P*<0.0001). Melanoma knowledge was positively associated with education level (*P*=0.002) and district of residence (*P*=0.004). Level of Concern was positively associated with age (*P*=0.026) and education level (*P*=0.041).

**Conclusions:**

There are gaps in melanoma knowledge and prevention practices in the study population. Further education on melanoma and its prevention should be specifically targeted to individuals of lower education levels as well as teenagers, such that protective behaviors for melanoma are adopted early in life.

**Supplementary Information:**

The online version contains supplementary material available at 10.1186/s12889-021-12324-0.

## Background

Skin cancer is the most common cancer worldwide [1]. Changes in solar ultraviolet (UV) radiation are anticipated due to stratospheric ozone depletion, climate change, and other factors such as absorption and scattering of aerosols and clouds, surface reflectivity, and solar activity [2]. These changes will result in increased exposure of the Earth’s surface to UV radiation from the end of the 20^th^ to the end of the 21^st^ century [2] and may also lead to increased incidence of skin cancers [3]. Melanoma, a prominent type of skin cancer, is responsible for most skin cancer-related deaths [4]. The global age-adjusted incidence rate of melanoma was five per 100,000 persons (95% CI, 4-7), while the age-adjusted mortality rate was one per 100,000 persons (95% CI, 0.7-1.0) in 2015 [5]. Various factors contribute to increased risk of melanoma, including UV radiation exposure, indoor tanning, light skin pigmentation, presence of atypical nevi and a higher number of nevi, personal and family history of melanoma, personal history of prostate cancer, consumption of alcohol, and chronic intake of certain medicines such as TNF-inhibitors and sildenafil [6–13]. Caffeinated coffee consumption and vitamin D intake are believed to be protective factors for melanoma [14, 15].

Melanoma is the 8^th^ most common cancer in males and the 10^th^ in females in Cyprus, which is an island in the Mediterranean with a population of 875,900 people in 2018 and a high UV index, [16, 17]. In 2012, the age-adjusted incidence rate of melanoma in Cyprus was 4.6 per 100,000 persons, while the age-adjusted mortality rate was 1.2 per 100,000 persons [18].

Over time, the age-adjusted incidence rates of melanoma in Cyprus have been increasing in both men and women; from 2.88 per 100,000 in 2002 to 4.46 per 100,000 in 2008 in males and from 3.56 per 100,000 to 4.61 per 100,000 in females [19]. The increase could partly be due to the year-round sunny weather. The population-weighted average daily ultraviolet level in Cyprus is higher compared to other countries in the Mediterranean region [20]. In addition, Cyprus represents a hotspot in climate change and is projected to face significant temperature increases [21] and potentially an increased melanoma risk by the end of this century [22, 23].

Both primary (wearing protective clothing, using sunscreen, and protecting oneself from UV exposure) and secondary (self-checking moles or having a health professional conduct a skin examination for skin cancer) forms of prevention are vital to the reduction of melanoma incidence and mortality rates [24]. The most effective way to decrease melanoma incidence is through the implementation of guidelines, public policies, and increased awareness and education regarding melanoma [25]. Studies have reported lack of awareness regarding the risk factors, signs and symptoms, and strategies for prevention of melanoma among the general population in Western countries [26–30], with a large portion of participants being unaware of melanoma and not performing self-examination for melanoma prevention.

The 2003 SunSmart project in the UK revealed low prevalence of melanoma prevention knowledge [26]. Another study in northern European countries found that 37.4%, 29.4%, 51.9%, and 32.1% of people were unaware of the increased melanoma risk after being sunburned in childhood in Denmark, Northern Ireland, Norway and Sweden, respectively. The same study revealed low prevalence of awareness of the risks of sunbed use in melanoma development and how mole changes reflect melanoma development [29]. Another study revealed that Australians had a significantly higher score of melanoma concern level, protective behavior, and knowledge compared to Scots [30].

In certain countries, the awareness of melanoma is growing both in the general population and among doctors, while some other countries are still lacking robust preventive strategies [24, 31, 32]. Cyprus does not have any melanoma screening or awareness programs in place. Considering the increasing incidence of melanoma in Cyprus and its high UV index environment, it is important to investigate the level of melanoma knowledge and protective behaviors in the general population. The ultimate goal is to set up evidence-based intervention programs to increase uptake of primary and secondary prevention strategies.

## Methods

### Study design and data collection

We conducted a cross-sectional study of melanoma knowledge, using a validated questionnaire [30], in Cyprus from October 2015 to April 2016. Six hundred participants, 18 years of age and older, from the districts of Nicosia, Larnaca, Paphos, Ammochostos, and Limassol were interviewed based on proportional quota sampling. Participants were recruited in public areas. Participants gave written consent by initialing the study information page and were either interviewed or they self-completed the survey in paper form. Approval for this study was obtained from the Cyprus National Bioethics Committee and Data Protection Office (Case Number: 3.28.399).

### Assessment tool and measures

A previously validated and published questionnaire was used in this study. Permission to use the questionnaire was obtained from the authors of the published study on melanoma knowledge in Australia and Scotland that utilized this questionnaire [30]. The questionnaire was translated into Greek and then back-translated to English by two different individuals to verify translation accuracy. The questionnaire consisted of 47 questions designed to assess participants' demographics, knowledge of melanoma and its risk factors, attitudes toward relevant health practices, and protective behaviors. Demographic categorical variables included sex, age, district (i.e., area of residence), and education level. Based on the answers to specific questions, a summary score was calculated for each of the following outcomes: personal risk, level of concern, protective behavior, and melanoma knowledge. For melanoma knowledge, each correct answer received a score of 1, whereas incorrect answers received a score of -1, and "Don't know" answers received a score of 0. There were 18 questions for the melanoma knowledge section. For the other outcomes (level of concern, protective behavior, and personal risk), answers reflecting those outcomes received higher scores than answers that did not reflect those outcomes. For example, one of the questions included in the summary score for personal risk was the number of times someone was sunburnt. An answer of "0 times" received a score of 0, an answer of "1-2 times" received a score of 2 and an answer of "3 or more times" received a score of 4. The total questions for the personal risk section were six while those for the protective behaviour section were nine.

Outcomes included protective behavior, which was assessed by asking participants about the frequency with which they check themselves for moles, and if they ever take measures to protect their skin from the sun; score ranging from -3 to +14. Knowledge of melanoma was another outcome investigated. It was assessed by asking participants about their knowledge of specific types of skin cancer, risk factors of melanoma, if they know of a family member or have an acquaintance with melanoma, and the body parts where melanoma most commonly develops in men and women; score ranging from -6 to +18). Personal risk was another outcome that was assessed based on questions relating to skin, hair and eye color as well as skin characteristics such as number, size and shape of moles, as well as number of times skin was sun damaged (i.e., individuals’ risk for developing melanoma; scores ranged from 0-20). Finally, level of concern was assessed by asking questions regarding skin self-examination, or examination by a health care professional for the appearance of abnormal moles (score ranged from 0-10).

### Statistical Analysis

Statistical analysis was performed using the Statistical Analysis System (SAS) software. Multiple Linear Regression analysis was conducted to investigate the effect of potential predictors on the various summary scores from the questionnaire. Predictors included sex, age, educational level, and district, and the outcomes were the summary scores of Level of Concern (scored 0 to 10), Protective Behavior (scored –3 to 14), Melanoma Knowledge (scored –6 to 18), and Personal Risk (scored 0 to 20).

The study examined associations of demographic variables, physical traits, and family history of skin cancer with levels of melanoma awareness and protective behaviors. Multiple linear regression was used to determine the association of sex, age, district, and level of education with the summary scores of personal risks, protective behavior, melanoma knowledge, and level of concern. An ANOVA test was done to evaluate the association of the number of skin moles with personal risk. When summary scores were not normally distributed (e.g., Level of Concern), a transformation with the logarithm was attempted, and normality of the log-transformed level of concern was further tested. It appears to have a moderate normal distribution. Therefore, multiple linear regression was also conducted to determine the association between demographic variables and log-transformed level of concern. Significance was set at *P*<0.05.

## Results

### Demographics of participants

Of the 600 completed questionnaires collected, 282 (47%) of the participants were men and 318 (53%) were women. The breakdown of participants by age, district and education level is presented in Table 1. In summary, there was a fairly even distribution across ages, most participants came from Nicosia which is the largest district, and most participants have a Diploma/Bachelor’s degree (Table 1).

**Table 1:** Demographics of participants

Characteristic	Number ***N*** = 600 (100.00%)
**Sex**	
Male	282 (47.00)
Female	318 (53.00)
**Age (years)**	
18 - 24	85 (14.00)
25 - 29	78 (13.00)
30 - 34	59 (10.00)
35 - 39	67 (11.00)
40 - 44	49 (8.00)
45 - 49	51 (9.00)
50 - 54	56 (9.00)
55 - 59	36 (6.00)
60 +	118 (20.00)
**District**	
Nicosia	227 (38.00)
Larnaca	114 (19.00)
Limassol	158 (26.00)
Paphos	63 (11.00)
Ammochostos	38 (6.00)
**Educational level**	
Elementary/Middle School	60 (10.00)
High school	202 (34.00)
Diploma/Bachelor's degree	234 (39.00)
Master's degree/PhD	103 (17.00)

### Primary and Secondary prevention practices for melanoma

When asked whether they check their skin for moles, 350 (59%) participants answered yes. Of those participants that check their skin for moles, 185 (31.19 %) check more than one time per month, 125 (21.07%) check their skin for moles monthly, and 40 (6.74%) check once or twice a year. In addition, 524 (87.00%) participants answered that they protect their skin from the sun by using sunscreen, wearing hats, or wearing other protective clothing during summer holidays. However, 340 (57.00%) of the participants answered that they don't take measures to protect their skin from the sun during the non-holiday period (Supplemental Table 1).

### Knowledge of melanoma and risk factors

When participants were asked which types of skin cancer they are aware of, 309 (51.50%) were aware of malignant melanoma only, and 234 (39.00%) were not aware of any skin cancer. Only 28 (4.67%) were aware of both malignant melanoma and squamous cell carcinoma, 4 (0.67%) were aware of melanoma and basal cell cancer, 18 (3.00%) were aware of all types, 2 (0.33%) were only aware of squamous cell carcinoma, and 1 (0.17%) was aware of both basal and squamous cell carcinoma (Table 2).

**Table 2:** Participants’ melanoma knowledge

Knowledge assessment	Number ***N*** = 600 (%)
**Types of skin cancers**	
Melanoma only	309 (51.50)
Melanoma and squamous cell cancer (SCC) only	28 (4.67)
Melanoma and basal cell cancer (BCC) only	4 (0.67)
SCC and BCC only	1 (0.17)
SCC only	2 (0.33)
Melanoma, SCC, BCC	18 (3.00)
None	234 (39.00)
Missing	4 (0.67)
**Site of skin cancer in men**	
Face, Legs, and other parts	206 (34.33)
Back	104 (17.33)
Do not know	288 (48.00)
Missing	2 (0.33)
**Site of skin cancer in women**	
Legs	57 (9.50)
Face, back, and other parts	223 (37.17)
Do not know	318 (53.00)
Missing	2 (0.33)
**Being aware of family members with a history of skin cancers**	
Yes	21 (3.50)
No	443 (73.83)
Do not know	136 (22.67)
**Being aware of non-family members with a history of skin cancers**	
Yes	61 (10.17)
No	378 (63.00)
Do not know	153 (25.50)
Missing	8 (1.33)

*BCC* Basal cell carcinoma, *SCC*, Squamous cell carcinoma, BCC and SCC are types of skin cancers.

Furthermore, most participants were not aware that the back is the most common site for melanoma in men: 288 (48.00%) of participants stated that they did not know the answer, 206 (34.33%) believed it was the face, legs, or another body part, and only 104 (17.33%) knew it was the back. Similarly, most participants were not aware that the legs are the most common site of melanoma in women: 318 (53.00%) stated that they did not know the answer, 223 (37%) believed it was the face, back, or another part of the body, and only 57 (9.50%) knew it was the legs (Table 2).

With regards to personal or family history of melanoma, 443 (73.83 %) did not have a family member diagnosed with malignant melanoma, 21 (3.50%) reported to have a family member with malignant melanoma, and 136 (22.67%) were not aware whether they had someone in their family with malignant melanoma. In addition, participants were asked if they knew someone else (other than family members) who had malignant melanoma. 378 (63.00%) did not know anyone in social circles with malignant melanoma, 61 (10.17%) knew someone with malignant melanoma, and 153 (25.50%) were not aware whether someone they knew had malignant melanoma (Table 2).

When asked about risk factors for melanoma, 401 (68.08%) of participants knew that having many moles was associated with increased risk for melanoma. In addition, 397 (67.75%) of participants knew that having a fair complexion is associated with increased risk for melanoma, 527 (88.42%) knew that getting sunburned is associated with increased risk for melanoma, and 503 (84.54%) knew that prolonged exposure to the sun is associated with increased risk for melanoma. However, most participants were not aware that having light-colored eyes or red/fair hair is associated with an increased risk for melanoma (Table 3).

**Table 3:** Participants’ knowledge of melanoma risk factors

Risk factors of skin cancers	Yes	No	Total
	Number (%)	Number (%)	Number (%)
Having many moles	401 (68.08)	188 (31.92)	589 (100.00)
Follow special diet	332 (57.43)	246 (42.56)	578 (100.00)
Having light skin	397 (67.75)	189 (32.25)	586 (100.00)
Drinking alcohol regularly	260 (44.91)	319 (55.09)	579 (100.00)
Sunburned	527 (88.42)	69 (11.58)	596 (100.00)
Extensive sun exposure	503 (84.54)	92 (15.46)	595 (100.00)
Smoking	190 (32.65)	392 (67.35)	582 (100.00)
Having blue eyes	45 (7.77)	534 (92.23)	579 (100.00)
Having green eyes	32 (5.53)	547 (94.47)	579 (100.00)
Having red hair	40 (6.92)	538 (93.08)	578 (100.00)
Having blonde hair	73 (12.72)	501 (87.28)	574 (100.00)
Having dark hair	301 (52.62)	271 (47.38)	572 (100.00)

### Association of Demographic Variables with Personal Risk, Protective Behavior, Melanoma Knowledge and Level of concern

The mean personal risk score for the entire population was 8.49, indicating that the population is at medium risk for melanoma based on these external features. The personal risk score for age was highest for participants in the range of 50 – 59 years (mean score of 9.28), whereas lowest for those 60 years and above (mean score of 7.44) (*p*=0.012). The personal risk score of melanoma increases with increasing level of education, making those with graduate (i.e., MSc/PhD) education to have the highest score (mean score 9.25) (*p*=0.027). When assessing the personal risk score based on the district where individuals reside, residents of Ammochostos (mean score 7.68) and Paphos (mean score 7.27) had lower scores compared to the other districts; Nicosia (mean score 8.64), Limassol (mean score 8.85), and Larnaca (mean score 8.64) (*p*=0.015). (Table 4)

**Table 4:** Mean scores of personal risk, protective behavior, melanoma knowledge and level of concern for different predictors

Outcome variables	Demographics	Mean (± SD)	***P***-value
**Personal risk**			
	**Age**		**0.012**
	18 – 24	8.20 (**±** 3.23)	
	25 – 29	9.04 (**±** 3.30)	
	30 – 34	8.83 (**±** 2.84)	
	35 – 39	8.49 (**±** 3.30)	
	40 - 44	8.96 (± 3.20)	
	45 – 49	8.80 (± 2.49)	
	50 – 54	8.84 (± 3.60)	
	55 -59	9.28 (± 3.23)	
	60 +	7.44 (± 3.25)	
	**Education**		**0.027**
	Elementary/Mid	7.27 (± 3.56)	
	High School	8.47 (± 3.43)	
	Diploma/BSC	8.48 (± 2.90)	
	MSc/PhD	9.25 (± 3.13)	
	**District**		**0.015**
	Nicosia	8.64 (± 3.40)	
	Larnaca	8.64 (± 2.84)	
	Limassol	8.85 (± 2.90)	
	Paphos	7.27 (± 3.32)	
	Ammochostos	7.68 (± 3.94)	
	**Number of moles**		**<.0001**
	None	5.36 (± 2.93)	
	Up to 20 moles	8.28 (± 2.85)	
	> 20 moles	10.85 (± 2.64)	
**Protective Behavior**			
	**Age**		0.581
	18 – 24	6.58 (± 2.37)	
	25 – 29	6.67 (± 2.47)	
	30 – 34	6.20 (± 2.49)	
	35 – 39	6.25 (± 2.68)	
	40 - 44	5.61 (± 2.35)	
	45 – 49	6.06 (± 2.53)	
	50 – 54	6.46 (± 2.68)	
	55 -59	6.33 (± 2.57)	
	60 +	6.39 (± 2.59)	
	**Education**		**0.016**
	Elementary/Mid	5.72 (± 2.53)	
	High School	6.03 (± 2.39)	
	Diploma/BSC	6.73 (± 2.52)	
	MSc/PhD	6.37 (± 2.68)	
	**District**		**<.0001**
	Nicosia	5.79 (± 2.31)	
	Larnaca	6.69 (± 2.40)	
	Limassol	6.25 (± 2.86)	
	Paphos	7.17 (± 2.39)	
	Ammochostos	7.37 (± 2.06)	
**Melanoma Knowledge**			
	**Age**		0.084
	18 – 24	6.72 (± 3.15)	
	25 – 29	7.54 (± 3.17)	
	30 – 34	7.37 (± 3.42)	
	35 – 39	6.78 (± 4.07)	
	40 - 44	6.65 (± 4.01)	
	45 – 49	8.31 (± 3.75)	
	50 – 54	7.57 (± 3.31)	
	55 -59	6.78 (± 3.16)	
	60 +	6.55 (± 3.45)	
	**Education**		**0.002**
	Elementary/Mid	5.88 (± 3.35)	
	High School	6.76 (± 3.35)	
	Diploma/BSC	7.39 (± 3.52)	
	MSc/PhD	7.83 (± 3.68)	
	**District**		**0.004**
	Nicosia	6.56 (± 3.33)	
	Larnaca	7.46 (± 3.61)	
	Limassol	7.82 (± 3.51)	
	Paphos	6.81 (± 3.32)	
	Ammochostos	6.55 (± 4.12)	
**Level of concern**			
	**Age**		**0.026**
	18 – 24	2.66 (± 1.94)	
	25 – 29	3.15 (± 2.03)	
	30 – 34	3.85 (± 2.28)	
	35 – 39	3.49 (± 2.38)	
	40 - 44	3.14 (± 1.90)	
	45 – 49	3.65 (± 2.38)	
	50 – 54	3.88 (± 2.17)	
	55 -59	3.83 (± 2.75)	
	60 +	3.32 (± 2.26)	
	**Education**		**0.041**
	Elementary/Mid	3.32 (± 2.62)	
	High School	3.13 (± 2.16)	
	Diploma/BSC	3.42 (± 2.20)	
	MSc/PhD	3.79 (± 2.18)	
	**District**		0.144
	Nicosia	3.56 (± 2.47)	
	Larnaca	3.67 (± 2.14)	
	Limassol	3.02 (± 2.00)	
	Paphos	3.16 (± 2.08)	
	Ammochostos	3.18 (± 2.05)	

Sex was not shown in the table because it is not statistically significant for personal risk, protective behavior, melanoma knowledge, and level of concern. 95% CI of the mean is significant if there is no overlap with corresponding confidence intervals of the same variables. Elementary/Mid=Elementary/middle school.

Protective behavior was significantly associated with educational level (*P*=0.016), with higher scores in participants with Diploma/Bachelor's degrees (mean score=6.73) and Master's Degrees/Ph.D. (mean score=6.37) compared to Elementary School/Middle School (mean score=5.71) and High School (mean score=6.08). Moreover, the district where participants lived was significantly associated with protective behavior (*P*<0.0001), with inhabitants of Ammochostos (mean score=7.37) and Paphos districts (mean score=7.17) scoring the highest for protective behavior compared to inhabitants of Larnaca (mean score=6.69), Limassol (mean score=6.24), and Nicosia (mean score=5.79). In contrast, age (*P*=0.581) and sex (*P*=0.378) were not significantly associated with protective behavior (Supplemental Table 1).

Melanoma knowledge was significantly associated with education level (*P*=0.002), with higher scores in participants with higher education level, such as Diploma/Bachelor's Degree (mean score= 7.39) and Master's Degree/Ph.D. (mean score= 7.83) compared to Elementary School/Middle School (mean score=5.88) and High School (mean score=6.73). Districts where participants lived was significantly associated with melanoma knowledge (*P*=0.004), with residents from Limassol (mean score 7.82) and Larnaca (mean score 7.46) with the highest knowledge. However, age (*P*=0.084), and sex (*P*=0.388) were not associated with melanoma knowledge (Table 4).

**Fig. 1:**
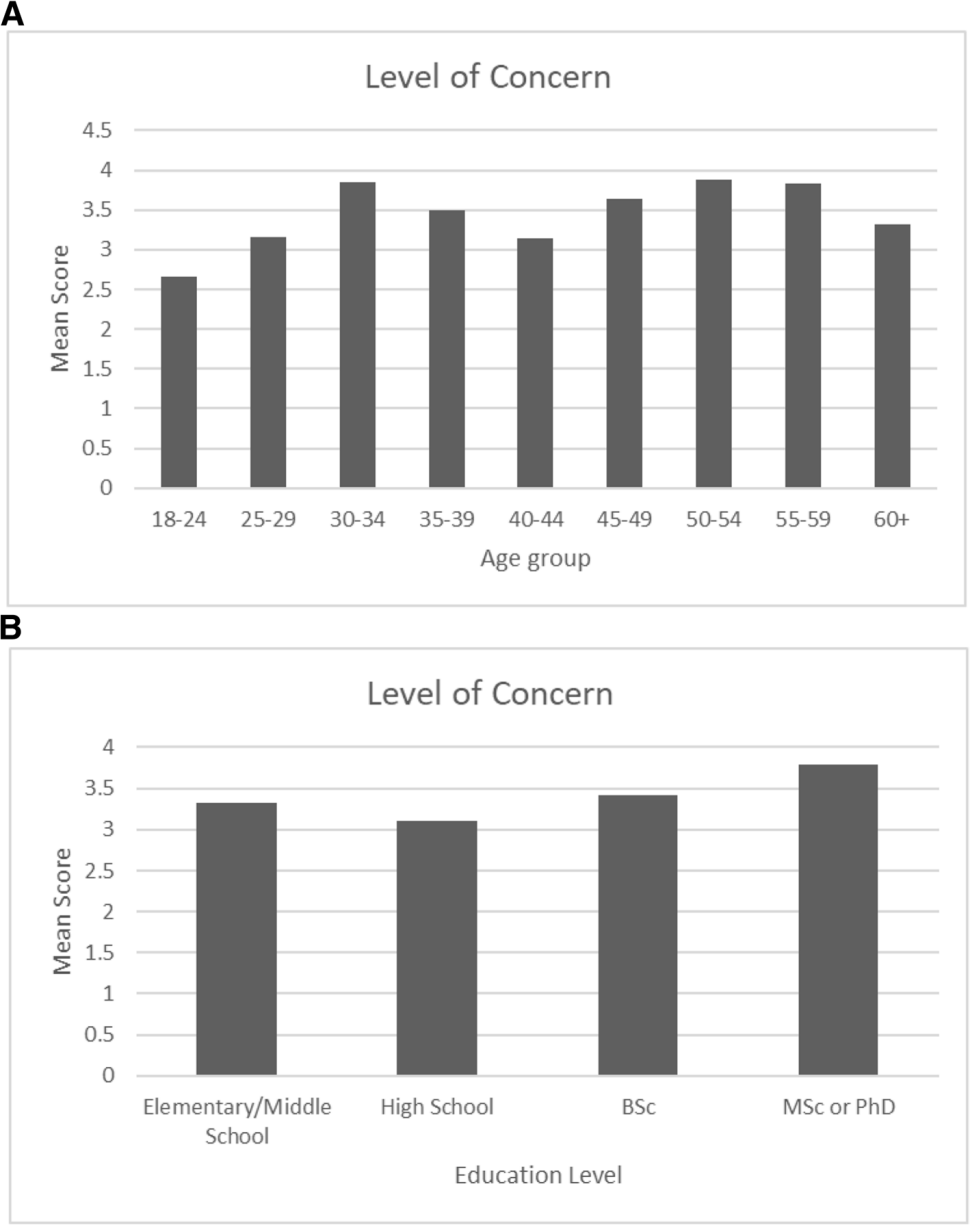
The figure shows the mean scores for level of concern in (**A)**. individuals of different age groups and (**B)**. in individuals of different levels of education (**A**)

Level of Concern was significantly associated with age (*P*=0.026) and education level (*P*=0.041), with higher levels of concern among older age groups and participants with higher levels of education (Table 4). More specifically, the youngest age group, 18-24, had the lowest mean score for level of concern (mean score=2.66), and participants with Master's degree/Ph.D. (mean score=3.79) had the highest levels of concern. Participants with Elementary/Middle School education had a mean score of 3.32, those with High School education had a mean score of 3.13, and those with Diploma/Bachelor's degree had a mean score of 3.42) (Fig. 1). Sex (*P*=0.431) and District (*P*=0.144) were not associated with the Level of Concern (Table 4).

### Predictors of personal risk, protective behavior, melanoma knowledge, and level of concern

Regarding personal risk, the odds of having a higher score for personal risk for those in the age group 55 – 59 was 3.63 times compared to the odds for those 18 – 24 years old. [OR^a^ – 3.63; *P*=0.045, 95% CI= 1.03, 12.68]. Furthermore, the odds of a higher score for personal risk for those with post-graduate education was 3.56 times the odds of those with elementary school education [OR^a^ – 3.56, *P*=0.029, 95% CI=1.14, 11.13]. Finally, the odds of having a higher score for personal risk for Paphos residents was 0.28 times the odds for Nicosia residents [ OR^a^ – 0.28, 95% CI = 0.11, 0.68, *P*=0.005) (Supplemental Table 2).

When we generated a multiple linear regression model, age group 40 – 44 years, graduate level education, and Larnaca/Limassol/Ammochostos residence were the only significant predictors of protective behavior. The odds of engaging in protective behaviors among age group 40 – 44 was 0.38 times the odds of the 18 – 24 age group. [OR^a^ = 0.38, 95% CI= 0.16, 0.91; *p*=0.031]. The odds of engaging in protective behaviors among those with graduate education and that undergraduate level education was 3.42 and 3.86 times the odds among those with elementary/middle school education, respectively [OR^a^ = 3.42; 95% CI = 1.40,8.25; *P*=0.007 and OR^a^ =3.86; 95% CI = 1.75, 8.50; *P*<0.001). Participants living in Ammochostos, Larnaca, and Paphos have higher odds of engaging in protective behaviors from melanoma than those in Nicosia (Supplemental Table 3).

Being 45 – 49 years old, having post-graduate education, and being a Larnaca and Limassol resident were the only significant predictors of melanoma knowledge in the multivariate linear regression models. For those 45-49 years old, the odds of having higher melanoma knowledge was 5.42 times the odds of those 18-24 years old [ OR^a^ – 5.42, *P*=0.006]. For those with post-graduate education, the odds of having higher melanoma knowledge was 6.89 times the odds of those with elementary school education. [OR^a^ – 6.89, *P*=0.002]. Participants who reside in the districts of Larnaca and Limassol have a higher likelihood of having a high melanoma knowledge score than those from the Nicosia district (Supplemental Table 4).

The predictors of level of melanoma concern were different compared to other outcome variables. In addition to post-graduate education and Limassol districts of residence, all the age groups were positively associated with a higher level of melanoma concern. Age group 50 – 54 has the highest odds with 1.25 times greater odds of having a higher level of melanoma concern than the 18 – 24 group. Those with post-graduate education (i.e., masters or Ph.D.) have 1.15 times higher chances of showing greater concern for developing melanoma than those with only elementary education. This is similar to previous findings for other outcome variables. However, only participants from the Limassol district have significantly higher odds of exhibiting a greater level of melanoma concern than their counterparts from Nicosia. [OR^a^ – 0.93, 95% CI – 0.87, 0.99, *P*=0.031] (Supplemental Table 5).

### The impact of skin mole multiplicity on melanoma knowledge and protective behavior

Most participants, 385 (65%), had up to 20 moles, followed by 137 (23%) with more than 20 moles and 74 (12%) with no moles. We hypothesized that people with more than 20 skin moles would be more aware of melanoma and consequently have higher scores in Protective Behavior and Melanoma Knowledge. None of the participants scored under 0 for melanoma knowledge (can range from -6 to +18) or protective behavior (can range from -3 to +14). Participants without skin moles had an average score of 6.72 for melanoma knowledge. Those with up to 20 skin moles scored an average of 7.18, and those with more than 20 skin moles scored an average of 7.07. Melanoma knowledge was not significantly different among these groups (ANOVA test, *P*=0.963).

Participant scores ranged from 0 to 12 for protective behavior. Participants without skin moles had an average score of 6.27 for protective behavior; those with up to 20 skin moles scored 6.71, and those with more than 20 skin moles scored 5.33. Protective behavior was highly significantly different among these groups, with the group with up to 20 skin moles showing much higher protective behavior than the other groups (ANOVA test, *P*< 0.0001) (Supplemental Figure 1).

## Discussion

The study addressed knowledge on melanoma and melanoma prevention in a population residing in a high-risk area with respect to UV exposure, one of the major risk factors for melanoma. With regards to primary prevention, 87.00% of the study participants reported protecting their skin from the sun during the summer holidays. However, 57.00% reported not taking measures to protect their skin from the sun during the non-holiday periods. This is alarming considering that Cyprus is sunny most days of the year and is a high UV index area with abundant solar radiation [33, 34]. With regards to secondary prevention, only 59.00% of study participants reported checking their skin for moles. This may be explained by the fact that 39.00% of study participants reported not being aware of any types of skin cancer.

Additionally, Cyprus is projected to have significant temperature increases from 1.0°C to 5.5°C by the end of the 21st century. These are higher than the projected global warming (1.8-4.0°C), with the largest warming over Southern Europe and the Mediterranean in summer [33, 34]. Thus, there is a need to raise awareness about melanoma and modes of prevention in this population. Interestingly, 73.83% of participants did not have a family member with melanoma, and 63.00% did not know anyone in their social circle with melanoma. This may potentially contribute to lower awareness of melanoma.

The study also addressed the knowledge of participants regarding melanoma risk factors. The majority of participants were aware that getting sunburnt and prolonged exposure to the sun are risk factors for melanoma (88.42% and 84.54%, respectively). However, fewer participants (68.08%) were aware that having many moles can be a risk factor for melanoma. Moreover, the majority of participants were not aware of the body features (e.g., blue and green eyes, red and fair hair) associated with an increased risk for melanoma. Hence, there is a need to raise awareness about all the risk factors for melanoma so that high-risk groups can implement primary and secondary melanoma prevention.

The study assessed personal risk for melanoma which included questions related to skin, hair, and eye color as well as skin characteristics such as number, size, and shape of moles and the number of times skin was sun-damaged. The study participants scored 8.49 on a scale with a maximum score of 20. Thus, the study population is one of medium personal risk, which is in line with the Southern position of the island and higher prevalence of darker hair and eyes. Age was a predictor of personal risk; 55-59-year-old individuals had higher odds of having a higher personal risk score compared to 18-24-year-old individuals, perhaps reflecting longer sun exposure of older individuals during their lifetime and consequently more skin damage and increased number of moles over the years. Individuals with graduate level education had higher odds of a higher personal risk score compared to individuals of elementary/middle school education. This may potentially be explained by a bigger awareness on what abnormal moles look like and in general better awareness of their personal risk. Finally, individuals of one district (Paphos) had lower odds of personal risk compared to individuals of other districts. This may reflect a higher prevalence of slightly darker skin color in that area and consequently lower personal risk.

Protective behavior was significantly associated with level of education and district. Participants with higher levels of education exhibited significantly higher level of protective behaviors. This suggests that education on melanoma may increase protective behavior. Inhabitants from Ammochostos and Paphos districts exhibited more protective behaviors than other districts, perhaps reflecting that these are districts by the sea with summer tourism as their main source of revenue [35]. Therefore, inhabitants of those districts spend more time outdoors and have learned to protect themselves from sun damage. Age, education and district of residence were predictors of protective behavior. Individuals 40-44 years old had lower odds of protective behavior compared to individuals 18-24 years old. Perhaps the younger generation has more exposure to melanoma prevention campaigns through the social media and hence tend to protect themselves more from sun exposure. Individuals with higher level of education have higher odds of protective behavior compared to those with lower education, suggesting that education may contribute to knowledge and consequent protective behavior. Finally, individuals residing in Ammochostos, Larnaca and Paphos had higher odds of protective behavior compared to individuals residing in Nicosia. Interestingly, Nicosia is a district inland whereas the others are by the sea. Inhabitants of areas by the sea may spend more time outdoors and consequently be more aware of sun damage and engage in protective behavior.

Melanoma knowledge was also significantly associated with educational level, education and area of residence. Individuals of ages 45-49 had higher odds of melanoma knowledge compared to individuals age 18-24 years old. The same was true for individuals with higher education, indicating that education has a positive impact on health awareness and knowledge. Finally, those from Larnaca and Limassol districts had higher odds of melanoma knowledge. Perhaps this may be due to the seaside location of these districts and more sun exposure of their residents.

Age was significantly associated with the level of concern, with the youngest age group reporting the lowest level of concern. Younger people may not consider skin cancer as seriously, thus requiring additional education. Older individuals had higher odds of having a higher level of concern compared to 18-24-year-old individuals. This may reflect the general tendency of people to be more mindful of their health as they get older and seek more medical attention. Individuals of higher education and also those residing in districts by the sea had higher odds of having a higher level of concern, further pointing to the importance of education and personal experience with sun exposure in prompting individuals to be concerned about melanoma.

The questionnaire we used was obtained from a similar study in Scotland that compared Scots to Australians [30]. Australians had higher levels of knowledge, level of concern and protective behavior compared to Scots, perhaps reflecting their higher exposure to the sun as well as the widely publicized thinning of the stratospheric ozone layer above Antarctica, resulting in increased exposure of Australians to UV-B radiation [36–38]. Interestingly, educational status was associated with level of concern, protective behavior, and knowledge in both the Scottish and Australian populations; a finding similar to our study’s findings. Another study conducted in Turkey, assessed awareness of skin cancer and prevention among university students [39]. The students exhibited lower prevention activities compared to the population of our study; 4.5% of the students practiced self-examination for skin cancer, whereas 58% of our study population reported checking their skin for moles. This difference may be attributed to the younger age of university students who probably do not think of cancer at this stage of their life. Interestingly, the youngest age group (18-24) had the lowest level of concern in our study.

Despite the low levels of melanoma knowledge in Cyprus, melanoma incidence continues to be slightly below the global incidence rate of 5 per 100,000 persons. However, behavioral changes in Cyprus that result in risky sun exposure behavior such as spending more time in the sun, less clothing cover (more skin exposed), and preference for a tan as well as more frequent, more intense, and longer lasting heat waves [40] are expected to increase melanoma incidence unless counteracted by protective behaviors, such as seeking shade, wearing protective clothing and sunglasses, and using sunscreens. Thus, melanoma-focused messages in summer campaigns, as well as messages regarding sun exposure in the non-holiday seasons may keep melanoma incidence low.

A limitation of our study was that participants were not randomly selected. Instead, they were approached in public places and asked to participate, which may have created selection bias since individuals with higher levels of melanoma concern may be more willing to answer the questionnaire. However, we did not observe high scores for melanoma knowledge or protective behavior in our study population. Another limitation is that there is a potential of self-reporting bias to those who self-completed the survey.

## Conclusions

Our study revealed some gaps in melanoma knowledge and prevention practices in individuals residing in a high-risk area for melanoma. Higher education level was significantly associated with higher scores in melanoma knowledge and prevention practices, suggesting that educational interventions regarding melanoma should be targeted to lower education level groups. In addition, interventions should be targeted to younger age groups such that melanoma prevention practices are implemented early on in life. Raising awareness about melanoma and methods of prevention should contribute to lower melanoma incidence and mortality.

A sunlight overloaded environment and climate change projections necessitate continued efforts to minimize the cost and morbidity of skin cancer in Cyprus. As efforts to address these health concerns are being implemented, the questionnaire could also gauge the effectiveness of the strategies.

## Supplementary Information

**Additional file 1: Supplemental Figure 1.** The figure shows the mean score for protective behavior in individuals with different number of moles. **Supplemental Table 1.** Primary and Secondary Prevention Practices for Melanoma. **Supplemental Table 2**. Predictors of personal risk as revealed by multiple linear regression. **Supplemental Table 3.** Predictors of protective behavior as revealed by multiple linear regression. **Supplemental Table 4.** Predictors of melanoma knowledge as revealed by multiple linear regression. **Supplemental Table 5**. Predictors of the level of concern as revealed by multiple regression.

## Data Availability

The datasets used and/or analyzed during the current study are available from the corresponding author on reasonable request.

## Abbreviation

SAS: Statistical Analysis System
