# Supplementary material for: Knowledge and Attitudes of Cypriots on Melanoma Prevention: Is there a Public Health Concern?
Source: BMC Public Health. 2022 Jan 8;22:53. doi: 10.1186/s12889-021-12324-0 (PMC8742933; doi:10.1186/s12889-021-12324-0)
Supplement: Supplementary file 1 — Additional file 1: Supplemental Figure 1. The figure shows the mean score for protective behavior in individuals with different number of moles. Supplemental Table 1. Primary and Secondary Prevention Practices for Melanoma. Supplemental Table 2. Predictors of personal risk as revealed by multiple linear regression. Supplemental Table 3. Predictors of protective behavior as revealed by multiple linear regression. Supplemental Table 4. Predictors of melanoma knowledge as revealed by multiple linear regression. Supplemental Table 5. Predictors of the level of concern as revealed by multiple regression. [file 12889_2021_12324_MOESM1_ESM.docx]

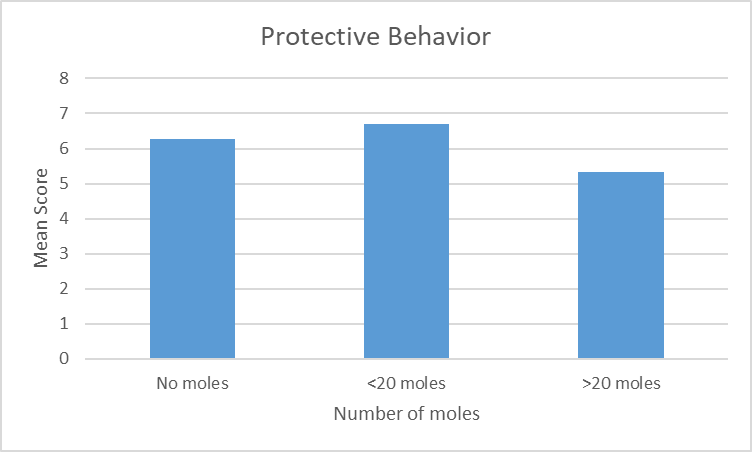


Supplemental Figure 1. The figure shows the mean score for protective behavior in individuals with different number of moles.
